# Effects of Water and Nitrogen Regulation on Alfalfa (*Medicago sativa* L.) Production Performance Through Optimization of Nitrogen Metabolism

**DOI:** 10.3390/plants15152380

**Published:** 2026-08-03

**Authors:** Mingzhu Wang, Hui Fan, Yubin Zhang, Minhua Yin, Yanxia Kang, Guangping Qi, Boda Li, Yuqing Yang

**Affiliations:** 1 College of Water Conservancy and Hydropower Engineering, Gansu Agricultural University, Lanzhou 730070, China; 1073324020379@st.gsau.edu.cn (M.W.); qigp@gsau.edu.cn (G.Q.); 1073324020375@st.gsau.edu.cn (B.L.); 1073324020394@st.gsau.edu.cn (Y.Y.); 2 Hydrology Bureau of Haihe River Water Conservancy Commission, Tianjin 300170, China; hhafan10@163.com; 3 Gansu Academy for Water Conservancy, Lanzhou 730000, China; shimano3300@126.com

**Keywords:** water and nitrogen regulation, nitrogen metabolism, crude protein content, yield, structural equation modeling, alfalfa

## Abstract

Water and nitrogen application rates directly affect crop yield formation and protein accumulation. Nitrogen metabolism, as a key physiological process linking water and nitrogen supply with crop growth, plays a critical role in regulating nitrogen uptake, transformation, and accumulation. However, the functional relationships among different nitrogen metabolism indicators in mediating the formation of high-quality and high-yield alfalfa under water–nitrogen regulation remain unclear. In this study, alfalfa (*Medicago sativa* L.) was subjected to four nitrogen application levels [N0 (0 kg·hm^−2^), N1 (80 kg·hm^−2^), N2 (160 kg·hm^−2^), N3 (240 kg·hm^−2^)] and four irrigation gradients [severe deficit (W0, 45–60% θ_f_), moderate deficit (W1, 55–70% θ_f_), mild deficit (W2, 65–80% θ_f_), and full irrigation (W3, 75–90% θ_f_), where θ_f_ represents field capacity]. The relationships between water–nitrogen regulation and alfalfa nitrogen metabolism and productive performance were analyzed. The results showed that (1) both irrigation amount and nitrogen application rate significantly affected the activities of leaf nitrate reductase (NR), glutamine synthetase (GS), glutamate synthase (GOGAT), and soluble protein (SP) content (*p* < 0.05). Aboveground nitrogen content (TN) initially increased and then decreased with increasing irrigation and nitrogen application, reaching its maximum under W2N2, with leaves being the primary site of aboveground nitrogen accumulation. (2) Under W2N2, alfalfa yield and crude protein accumulation (CP-a) both reached their maximum values, averaging 10.22 t·ha^−1^ and 1187.10 kg·ha^−1^, respectively. (3) Structural equation modeling (SEM) indicated that water–nitrogen regulation primarily influenced yield and CP-a formation through its effects on GS activity and TN. Based on these findings, a comprehensive evaluation model (GS–TN–Yield–CP-a) was constructed, and the preliminary suitable water–nitrogen regulation ranges for high-quality and high-yield alfalfa were identified as an irrigation amount of 69.4–85.9% θ_f_ and a nitrogen application rate of 102.6–222.3 kg·hm^−2^. These results can provide a theoretical basis for high-quality and high-yield alfalfa management in arid and semi-arid regions, but further verification through field experiments is still required.

## 1. Introduction

Water scarcity and insufficient soil nutrient supply in arid and semi-arid regions are important factors limiting forage production, and reasonable irrigation and nitrogen management play an important role in improving forage yield and quality. Alfalfa (*Medicago sativa* L.) is a high-quality perennial leguminous forage widely cultivated worldwide. Due to its high yield, palatability, and high crude protein content, it is hailed as the “king of forages” [1]. With its well-developed root system and strong ability to fix nitrogen biologically, alfalfa plays a vital role in improving soil structure, enhancing soil fertility, and promoting the sustainable development of agricultural ecosystems [2]. As the world’s most important origin of alfalfa germplasm resources and a major producer of alfalfa forage, China is the world’s second-largest alfalfa-growing country, with its cultivated area accounting for 15% of the global total [3]. Its alfalfa-growing regions are primarily distributed in the arid and semi-arid areas of Northwest China, including Inner Mongolia, Gansu, Ningxia, and Xinjiang [4]. However, the improvement of alfalfa productive performance and forage quality in this region has been severely constrained by water scarcity, poor soil fertility, and extensive management practices [5]. Therefore, optimizing water and nitrogen management practices and improving water and nitrogen use efficiency are of great significance for achieving high-yield, high-quality alfalfa cultivation.

Water and nitrogen are important factors affecting crop growth, development, yield improvement, and quality enhancement. Nitrogen metabolism is a key physiological process involving the assimilation, accumulation, and distribution of nitrogen in plants, and its metabolic level is directly related to nitrogen use efficiency and protein synthesis capacity [6]. Research has found that appropriate nitrogen application increased the activities of nitrate reductase (NR) and glutamine synthetase (GS), while excessive nitrogen application inhibited their activities [7]. Meanwhile, adequate nitrogen supply can alleviate the damage caused by water stress to nitrogen metabolism processes to a certain extent [8]. Conversely, under low nitrogen conditions, water stress reduces the activities of enzymes related to nitrogen metabolism in rice [9]. Furthermore, appropriate water and nitrogen regulation is conducive to maintaining a high level of nitrogen metabolism, thereby promoting the conversion of nitrogen from inorganic to organic forms, and enhancing nitrogen accumulation and protein synthesis capacity in plants [10]. Therefore, nitrogen metabolism is not only an important physiological process for nitrogen transformation and accumulation in plants, but also a key regulatory link through which water–nitrogen regulation affects crop productive performance. Studies have also found that mild deficit irrigation combined with appropriate nitrogen application can significantly increase crop biomass and yield [11]. Meanwhile, water–nitrogen interaction can increase nitrogen accumulation in plants [12]. Mohammadzadeh et al. [13] investigated the effects of different water and nitrogen conditions on tomato yield and quality at Ferdowsi University of Mashhad and found that adequate water and nitrogen had a synergistic promoting effect on fruit yield. Li et al. [14] studied the effects of different water and urea conditions on summer maize yield and leaf senescence characteristics at Shandong Agricultural University and found that nitrogen application rate significantly increased soluble protein content. Zhao et al. [15] conducted research in Northeast China on the comprehensive effects of water and nitrogen on tomato growth and water–nitrogen utilization and found that increasing irrigation and nitrogen fertilizer application improved tomato yield. Lu et al. [16] investigated the effects of water–nitrogen regulation on mung bean cultivation and found that the highest yield was achieved under the W3N1 treatment (60–80% of field capacity, 40 kg·ha^−1^). Kang et al. [17] studied the effects on alfalfa productivity in Zhangye City, Gansu Province and found that appropriate water–nitrogen combinations not only increased yield but also promoted crude protein accumulation and improved forage quality. Therefore, appropriate water–nitrogen regulation is conducive to maintaining a high level of nitrogen metabolism, promoting nitrogen accumulation in plants, and further improving crop productive performance.

The arid and semi-arid regions of northwestern China, as important alfalfa-producing areas in the country, possess advantages such as abundant light and heat resources and vast land resources. However, due to limited precipitation, high evapotranspiration, and restricted soil nutrient availability, improvements in alfalfa productive performance and forage quality remain constrained to a certain extent. Optimizing water–nitrogen management practices is therefore important for promoting high-yield and high-quality alfalfa production in this region. Nevertheless, existing studies have mostly focused on the effects of water–nitrogen regulation on individual physiological indicators or yield and quality, while the comprehensive relationships among nitrogen metabolism-related enzymes, related products, and productive performance remain poorly understood. Structural equation modeling (SEM) can simultaneously analyze the direct and indirect relationships among multiple variables and has been widely applied in studies of agroecology, physiological regulation, and crop production mechanisms [18]. We hypothesized that appropriate water–nitrogen combinations could regulate the activities of nitrogen metabolism-related enzymes in alfalfa leaves, thereby promoting the accumulation of related nitrogen metabolites and ultimately exerting positive effects on yield and crude protein accumulation. Furthermore, the relative contributions of individual nitrogen metabolism indicators to productive performance may differ under varying water–nitrogen conditions. In view of this, the present study employs SEM to analyze the effects of water–nitrogen regulation on nitrogen metabolism, yield, and crude protein accumulation in alfalfa, with the following objectives: (1) to clarify the effects of different water–nitrogen combinations on nitrogen metabolism-related enzymes, nitrogen metabolism-related products, and productive performance in alfalfa; (2) to elucidate the interrelationships among nitrogen metabolism indicators, yield, and crude protein accumulation using SEM, to identify key regulatory pathways, and to determine the appropriate water–nitrogen regulation ranges, thereby preliminarily providing a theoretical basis and practical guidance for optimizing water–nitrogen management models for alfalfa in the arid and semi-arid regions of northwestern China.

## 2. Results

### 2.1. Effects of Water and Nitrogen Regulation on the Enzyme Activities Related to Nitrogen Metabolism in Alfalfa

#### 2.1.1. Nitrate Reductase

**Figure 1:**
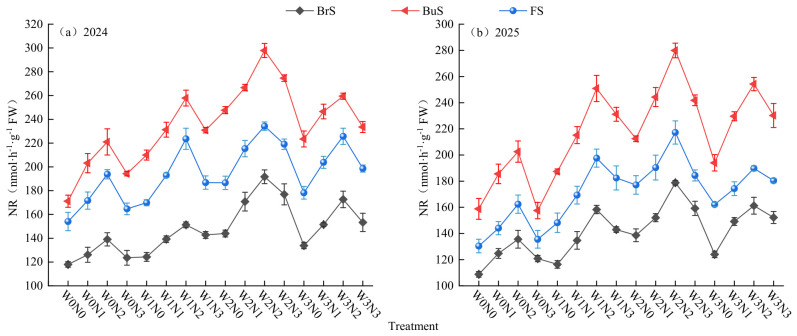
Effects of water–nitrogen regulation on nitrate reductase activity at different growth stages of alfalfa. Note: BrS, BuS, and FS represent branching, budding, and flowering stages, respectively. (**a**,**b**) represent nitrate reductase activity at each growth stage in 2024 and 2025, respectively.

Under different water–nitrogen treatments, leaf nitrate reductase (NR) activity in alfalfa exhibited a unimodal trend, first increasing and then decreasing across the branching, budding, and flowering stages, peaking at the budding stage. Meanwhile, irrigation amount, nitrogen application rate, and their interaction significantly affected NR activity (*p* < 0.05, Figure 1). During the two growing seasons, NR activity at the budding stage was higher than that at the flowering stage, while the lowest activity was observed at the branching stage. Under the same irrigation level, NR activity first increased and then decreased with increasing nitrogen application rate, reaching its maximum at the N2 level. Under the same nitrogen application rate, NR activity first increased and then decreased with increasing irrigation amount, and the W2 level was significantly higher than the W0, W1, and W3 levels. The highest NR activity was recorded under the W2N2 treatment, averaging 233.31 nmol·h^−1^·g^−1^ FW, which was 9.66–66.6% higher than that under the other treatments.

#### 2.1.2. Glutamine Synthase

**Figure 2:**
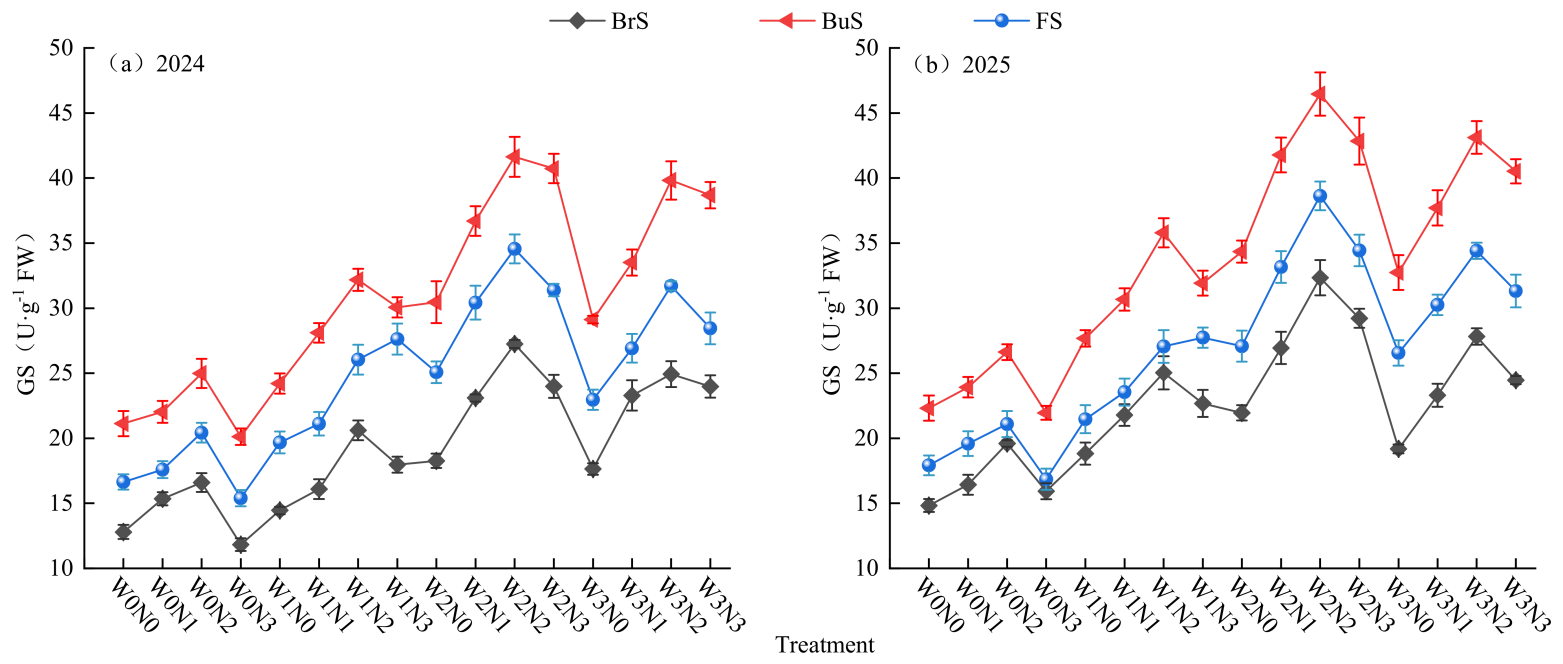
Effects of water–nitrogen regulation on glutamine synthetase activity at different growth stages of alfalfa. Note: BrS, BuS, and FS represent branching, budding, and flowering stages, respectively. (**a**,**b**) represent glutamine synthetase activity at each growth stage in 2024 and 2025, respectively.

Leaf glutamine synthetase (GS) activity of alfalfa exhibited a unimodal trend across growth stages, peaking at the budding stage, and was significantly influenced by irrigation amount, nitrogen application rate, and their interaction (*p* < 0.01, Figure 2). Under the same irrigation level, GS activity first increased and then decreased with increasing nitrogen application rate, reaching its maximum at the N2 level. Under the same nitrogen application rate, GS activity first increased and then decreased with increasing irrigation amount, following the order W2 > W3 > W1 > W0. The W2 treatment was on average 8.39–79.78% higher than the other treatments. Across all treatments, the maximum leaf GS activity occurred under the W2N2 treatment, with an average value of 36.81 U·g^−1^ FW.

#### 2.1.3. Glutamate Synthase

**Figure 3:**
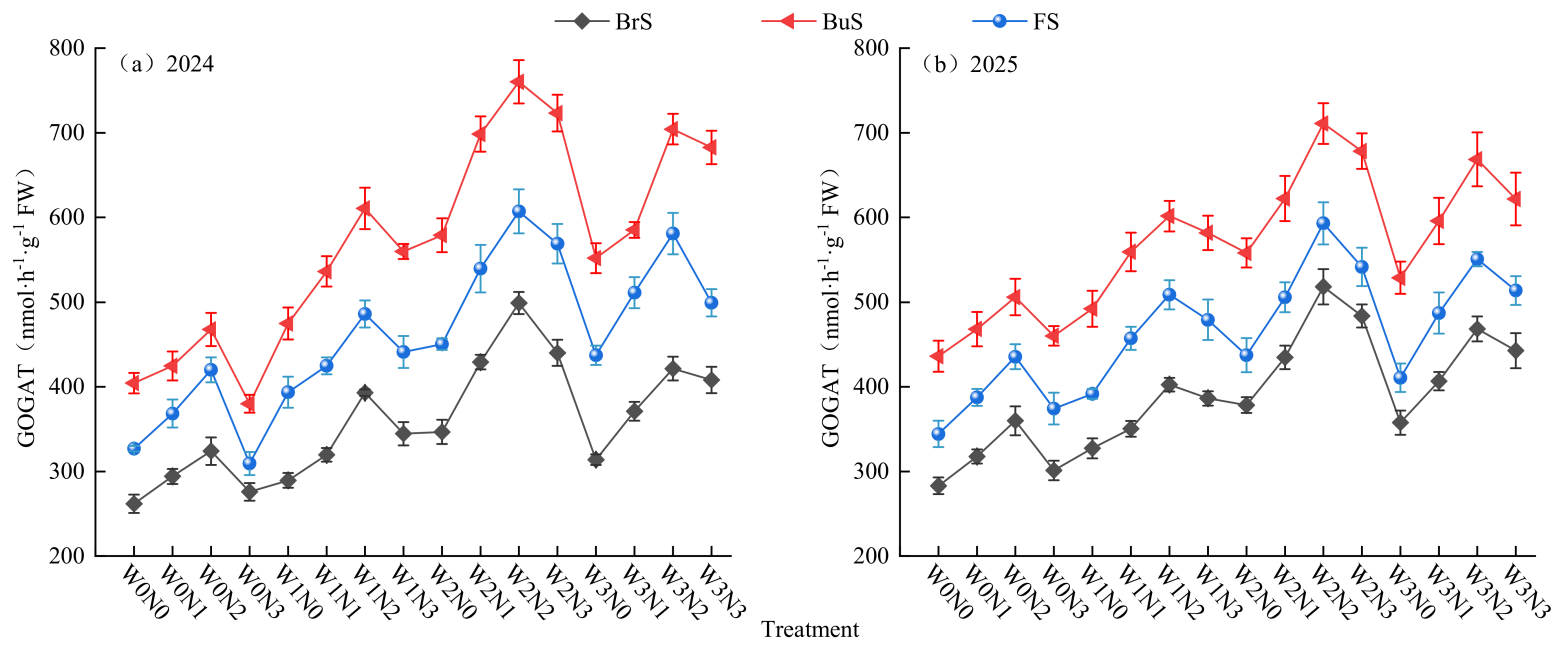
Effects of water–nitrogen regulation on glutamate synthase activity at different growth stages of alfalfa. Note: BrS, BuS, and FS represent branching, budding, and flowering stages, respectively. (**a**,**b**) represent glutamate synthase activity at each growth stage in 2024 and 2025, respectively.

Leaf glutamate synthase (GOGAT) activity of alfalfa exhibited a unimodal trend across growth stages, peaking at the budding stage, and was significantly influenced by irrigation amount, nitrogen application rate, and their interaction (*p* < 0.01, Figure 3). Under the same irrigation level, GOGAT activity first increased and then decreased with increasing nitrogen application rate, reaching its maximum at the N2 level. Under the same nitrogen application rate, GOGAT activity first increased and then decreased with increasing irrigation amount, following the order W2 > W3 > W1 > W0. The maximum GOGAT activity occurred under the W2N2 treatment, with an average value of 614.84 nmol·h^−1^·g^−1^ FW, which was, on average, 7.37–82.41% higher than that under the other treatments.

### 2.2. Effects of Water and Nitrogen Regulation on Nitrogen Metabolites in Alfalfa

#### 2.2.1. Soluble Protein Content

**Figure 4:**
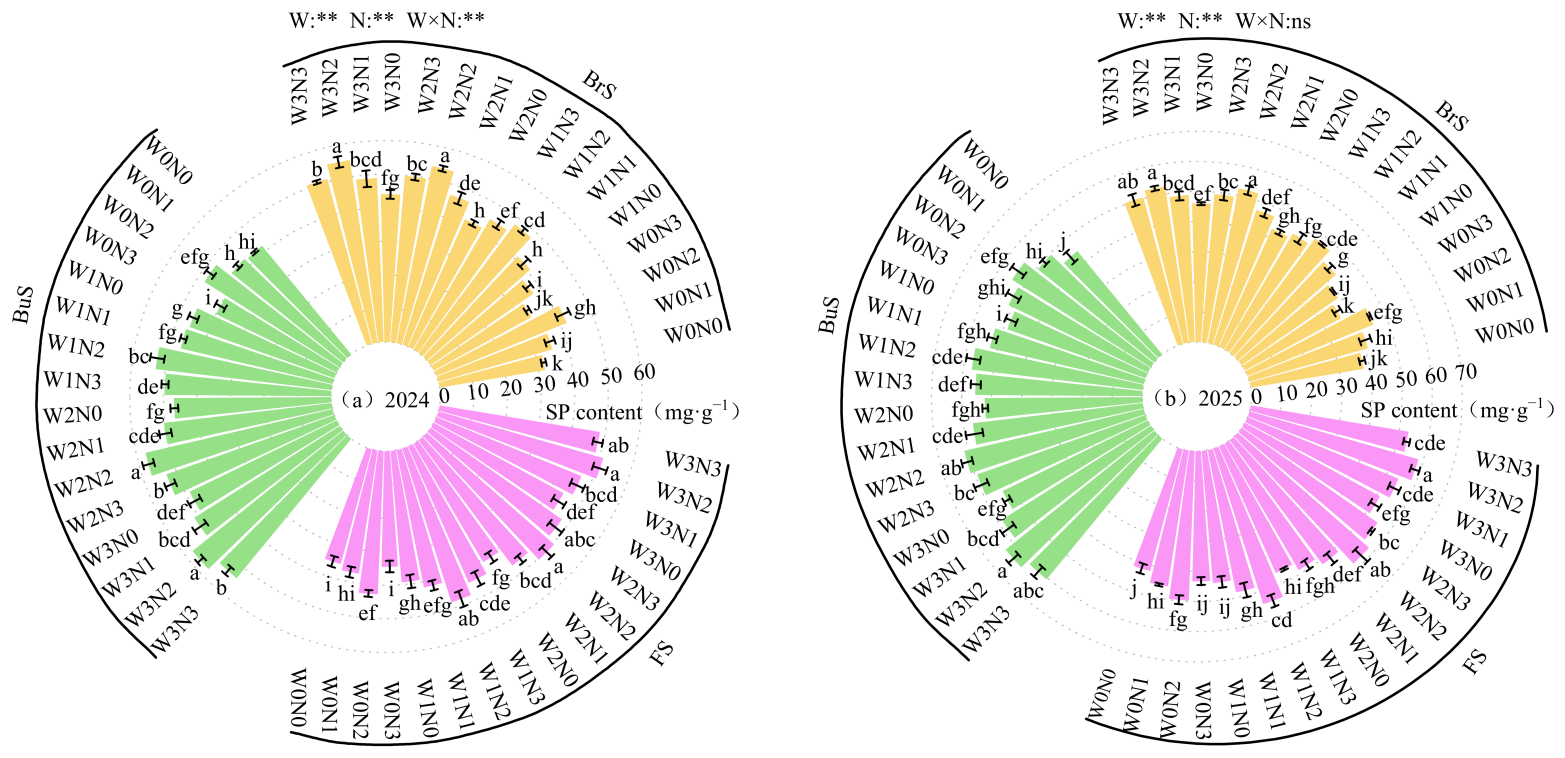
Effects of water–nitrogen regulation on soluble protein content at different growth stages of alfalfa. Note: BrS, BuS, and FS represent branching, budding, and flowering stages, respectively. ** indicate significance at *p* < 0.01, respectively; ns indicates not significant at *p* > 0.05. (**a**,**b**) represent soluble protein content at each growth stage in 2024 and 2025, respectively.

Leaf soluble protein (SP) content of alfalfa exhibited a unimodal trend across growth stages, peaking at the budding stage, and was significantly influenced by irrigation amount and nitrogen application rate at the branching, budding, and flowering stages (*p* < 0.01, Figure 4), while their interaction effect was significant only at the branching stage and at the budding stage in 2024. Under the same irrigation level, SP content first increased and then decreased with increasing nitrogen application rate, reaching its maximum at the N2 level, and was 9.06–20.20% higher than that under the other treatments on average. Under the same nitrogen application rate, SP content increased with increasing irrigation amount, following the order W3 > W2 > W1 > W0. The maximum SP content was recorded under the W3N2 treatment, followed by the W2N2 treatment, with values being 1.35–45.96% higher than those under the other treatments on average.

#### 2.2.2. Nitrogen Content and Accumulation in the Aboveground Parts

(1) Nitrogen content in the aboveground parts

**Figure 5:**
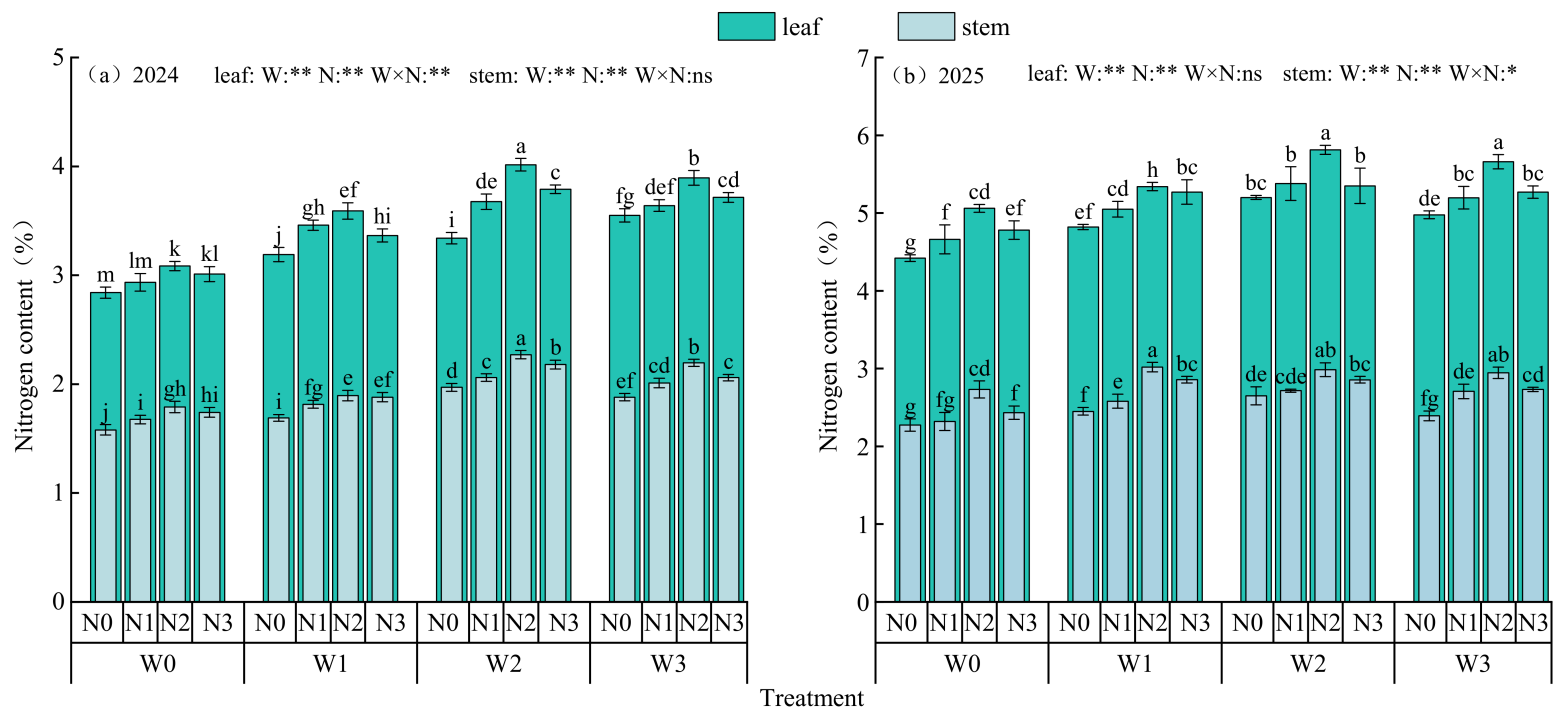
Effects of water–nitrogen regulation on aboveground nitrogen content of alfalfa at the flowering stage. Note: * and ** indicate significance at *p* < 0.05 and *p* < 0.01, respectively; ns indicates not significant at *p* > 0.05. (**a**,**b**) represent aboveground nitrogen content in 2024 and 2025, respectively. The nitrogen content of the aboveground parts includes the nitrogen content of the leaves and stems.

Nitrogen content of alfalfa was significantly influenced by irrigation amount and nitrogen application rate (*p* < 0.01, Figure 5), while their interaction effect was not significant on stem nitrogen content in 2024 and leaf nitrogen content in 2025. Under the same irrigation level, nitrogen content in both leaves and stems first increased and then decreased with increasing nitrogen application rate, reaching the maximum at the N2 level, with average increases of 5.46–18.74% and 5.54–17.05% compared with the other levels, respectively. Under the same nitrogen application rate, nitrogen content in leaves and stems overall first increased and then decreased with increasing irrigation level, reaching the maximum at the W2 level, with average increases of 1.58–19.89% and 3.44–19.92% compared with the other levels, respectively. Among all treatments, the highest aboveground nitrogen content was observed under the W2N2 treatment, followed by the W3N2 treatment, with average increases of 4.79–103.33% compared with the other treatments.

(2) Nitrogen accumulation in the aboveground parts

**Figure 6:**
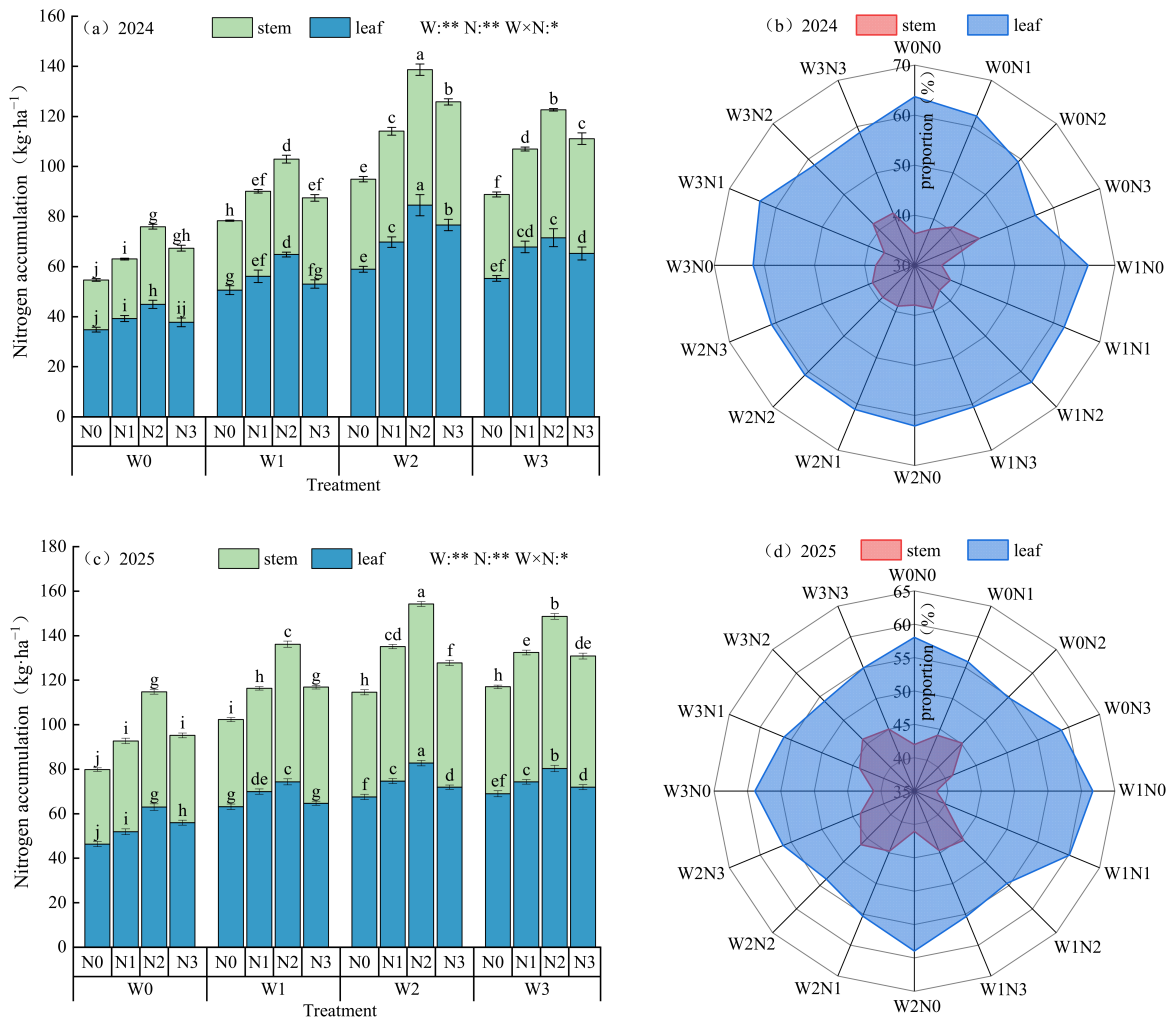
Effects of water–nitrogen regulation on aboveground nitrogen accumulation of alfalfa at the flowering stage. Note: * and ** indicate significance at *p* < 0.05 and *p* < 0.01, respectively; ns indicates not significant at *p* > 0.05. (**a**,**c**) represent nitrogen accumulation in 2024 and 2025, respectively. (**b**,**d**) represent the proportions of nitrogen accumulation in leaves and stems in 2024 and 2025, respectively. The nitrogen content of the aboveground parts includes the nitrogen content of the leaves and stems.

Nitrogen accumulation of alfalfa was significantly influenced by irrigation amount, nitrogen application rate, and their interaction (*p* < 0.05, Figure 6). Under the same irrigation level, nitrogen accumulation first increased and then decreased with increasing nitrogen application rate, reaching its maximum at the N2 level, with average increases of 14.29–36.41% compared with the other levels. Under the same nitrogen application rate, nitrogen accumulation first increased and then decreased or consistently increased with increasing irrigation level, generally reaching its maximum at the W2 level, with average increases of 5.38–60.22% compared with the other levels. Among all treatments, the highest nitrogen accumulation was observed under the W2N2 treatment, and the lowest under the W0N0 treatment, averaging 146.46 kg·ha^−1^ and 67.26 kg·ha^−1^, respectively. Further analysis of the composition of aboveground nitrogen accumulation revealed that across both years and all treatments, leaf nitrogen accumulation accounted for 54.85–63.15% of the total aboveground nitrogen accumulation, while stem nitrogen accumulation accounted for 36.85–45.15%.

### 2.3. The Effect of Water and Nitrogen Regulation on Alfalfa Yield

#### 2.3.1. Yield

**Figure 7:**
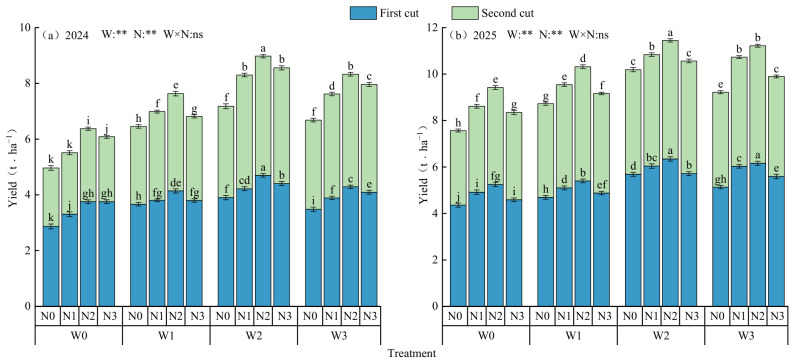
Effects of water–nitrogen regulation on alfalfa yield. Note: * and ** indicate significance at *p* < 0.05 and *p* < 0.01, respectively; ns indicates not significant at *p* > 0.05. (**a**,**b**) represent yield in 2024 and 2025, respectively.

Alfalfa yield increased with increasing planting year and was significantly influenced by irrigation amount and nitrogen application rate, while their interaction effect was not significant (*p* > 0.05, Figure 7). Under the same irrigation level, yield first increased and then decreased with increasing nitrogen application rate, reaching its maximum at the N2 treatment, with an average increase of 6.51–12.94% compared with the other levels. Under the same nitrogen application rate, yield first increased and then decreased with increasing irrigation level, reaching its maximum at the W2 level, following the order W2 > W3 > W1 > W0, and was on average 6.03–35.47% higher than the other levels. Among all treatments, the highest yield was recorded under the W2N2 treatment and the lowest under the W0N0 treatment, averaging 10.22 t·ha^−1^ and 6.27 t·ha^−1^, respectively.

#### 2.3.2. Crude Protein Content and Accumulation

(1) Crude protein content

**Figure 8:**
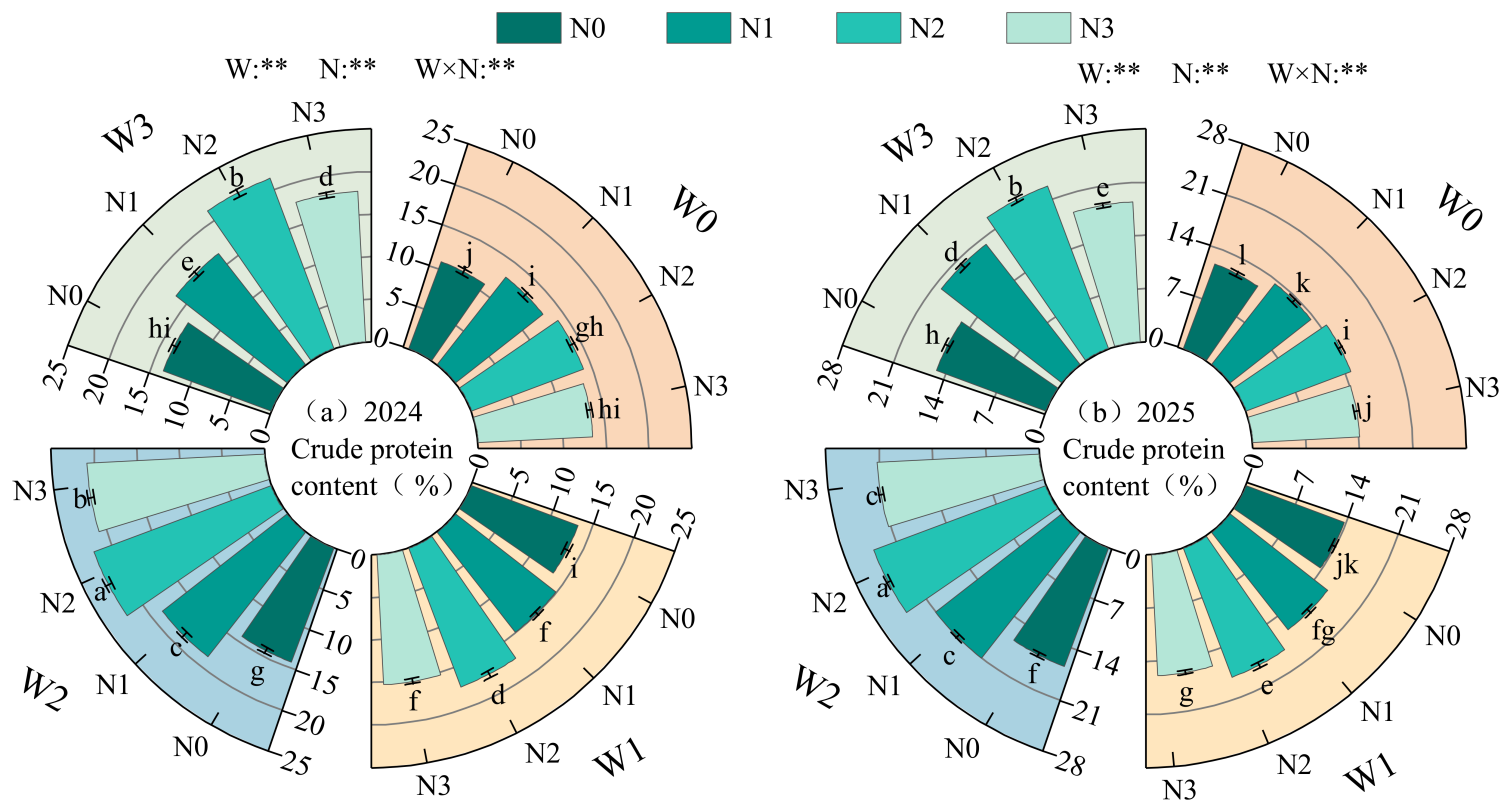
Effects of water–nitrogen regulation on crude protein content of alfalfa. Note: ** indicate significance at *p* < 0.01, respectively; ns indicates not significant at *p* > 0.05. (**a**,**b**) represent crude protein content in 2024 and 2025, respectively.

Crude protein content (CP) of alfalfa was highly significantly influenced by irrigation amount, nitrogen application rate, and their interaction (*p* < 0.01, Figure 8). Under the same irrigation level, crude protein content first increased and then decreased with increasing nitrogen application rate, reaching its maximum at the N2 level, with average increases of 12.14–41.38% compared with the other levels. Under the same nitrogen application rate, crude protein content first increased and then decreased with increasing irrigation amount, following the order W2 > W3 > W1 > W0, and was on average 8.97–50.40% higher than the other levels. Over the two years, the maximum CP content was recorded under the W2N2 treatment, and the minimum under the W0N0 treatment, averaging 23.13% and 11.38%, respectively.

(2) Crude protein accumulation

**Figure 9:**
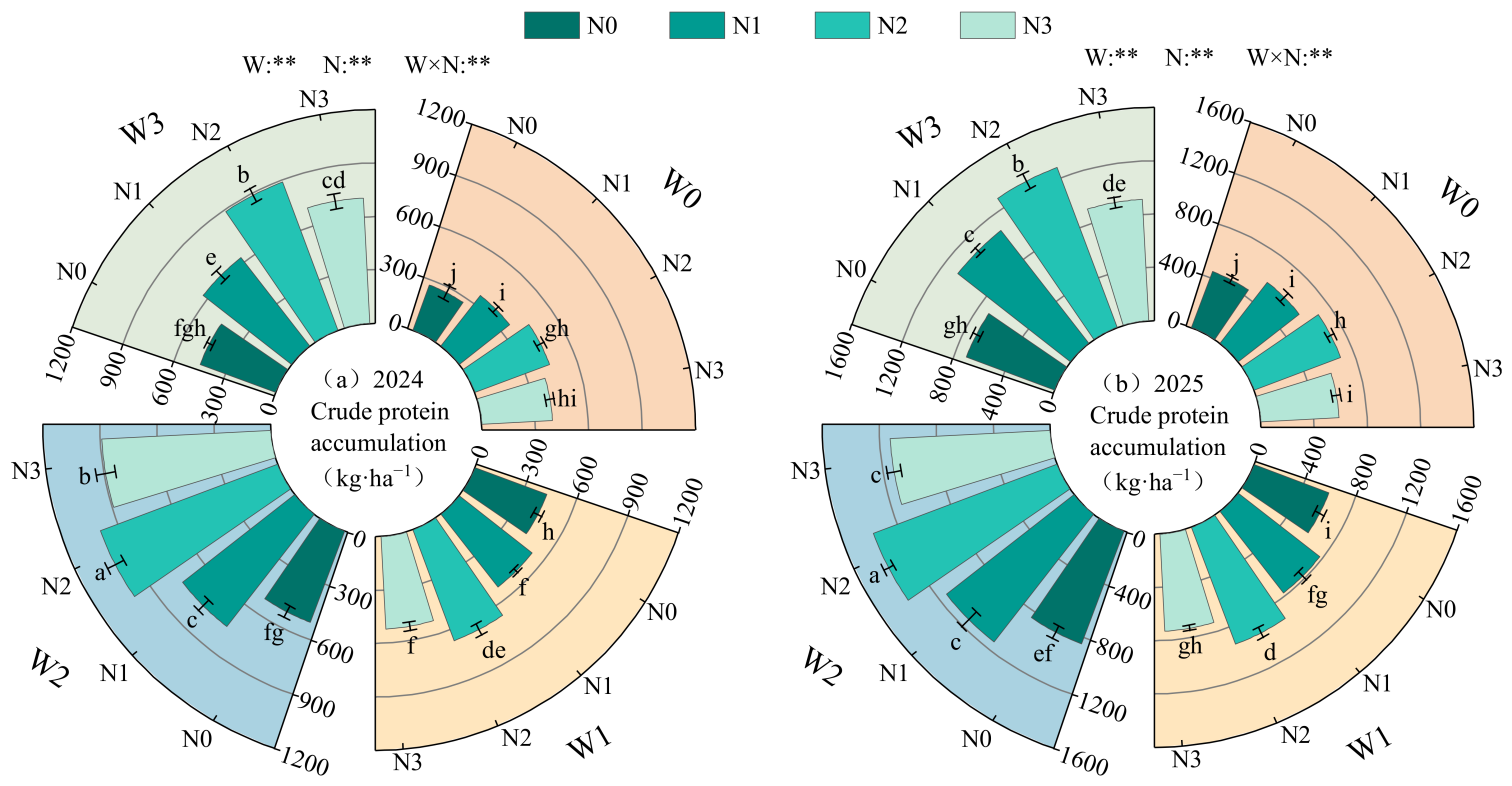
Effects of water–nitrogen regulation on crude protein accumulation of alfalfa. Note: ** indicate significance at *p* < 0.01, respectively; ns indicates not significant at *p* > 0.05. (**a**,**b**) represent crude protein accumulation in 2024 and 2025, respectively.

Crude protein accumulation (CP-a) of alfalfa was significantly influenced by irrigation amount, nitrogen application rate, and their interaction (*p* < 0.01, Figure 9). Under the same irrigation level, crude protein accumulation first increased and then decreased with increasing nitrogen application rate, reaching its maximum at the N2 level, with average increases of 19.74–72.47% compared with the other levels. Under the same nitrogen application rate, crude protein accumulation first increased and then decreased with increasing irrigation level, reaching its maximum at the W2 level, following the order W2 > W3 > W1 > W0, and was on average 15.29–104% higher than the other levels. Among all treatments, the maximum crude protein accumulation was recorded under the W2N2 treatment, and the minimum under the W0N0 treatment, averaging 1187.10 kg·ha^−1^ and 359.70 kg·ha^−1^, respectively.

### 2.4. Relationships Among Enzymes and Metabolites Involved in Alfalfa Nitrogen Metabolism and Production Performance Under Water and Nitrogen Limitation

#### 2.4.1. Structural Equation Modeling

**Figure 10:**
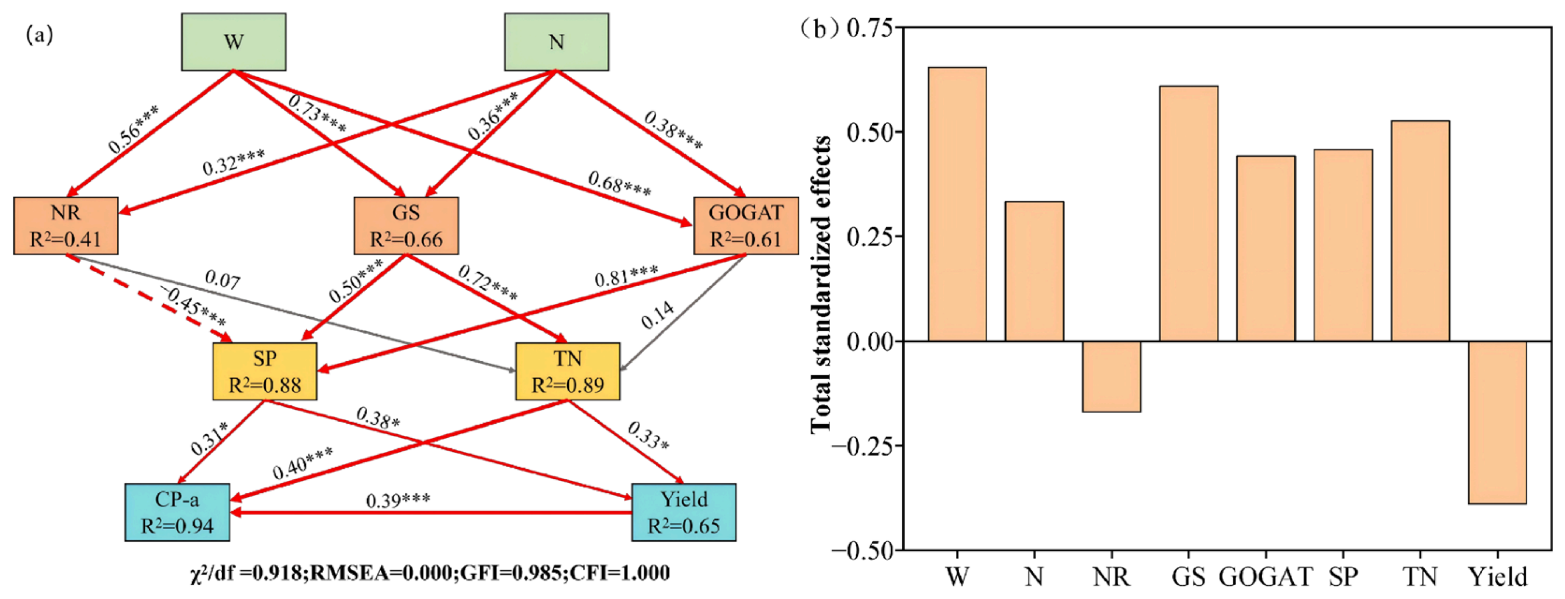
Structural equation model (SEM). Note: W: irrigation amount (%θ_f_); N: nitrogen application rate (kg·hm^−2^); NR: nitrate reductase activity (nmol·h^−1^·g^−1^ FW); GS: glutamine synthetase activity (U·g^−1^ FW); GOGAT: glutamate synthase activity (nmol·h^−1^·g^−1^ FW); SP: soluble protein content (mg·g^−1^); TN: total plant nitrogen content (%); Yield (t·ha^−1^); and CP-a: crude protein accumulation (kg·ha^−1^). Red solid lines and red dashed lines indicate significant positive and negative relationships, respectively; gray solid lines indicate non-significant relationships; and line thickness indicates the strength of the effect. *, and *** indicate significance at *p* < 0.05 and *p* < 0.001, respectively; ns indicates not significant at *p* > 0.05. R^2^ values within boxes indicate the proportion of variance explained for each variable. (**a**) structural equation model diagram; (**b**) total standardized effects.

To further elucidate the relationships among nitrogen metabolism-related indicators and productive performance under water–nitrogen regulation, a structural equation model (SEM) was constructed to quantitatively analyze the direct and indirect effects of irrigation amount and nitrogen application rate on various indicators (Figure 10a). The fit indices showed that χ^2^/df = 0.918, which was below 3, indicating a good model fit. GFI = 0.985 and CFI = 1.000, both greater than 0.9, indicating high explanatory power and stability of the model. RMSEA = 0.000, which was below 0.08, indicating a small approximation error. The SEM results showed that under different water–nitrogen regimes, the coefficient of determination (R^2^) for yield was 0.65, indicating that SP and TN together explained 65% of the yield variation, with SP being the main direct influencing factor, with a standardized path coefficient of 0.38. For crude protein accumulation (CP-a), the R^2^ was 0.94, indicating that SP, TN, and yield together explained 94% of the variation, with TN and yield being the main direct influencing factors, with standardized path coefficients of 0.40 and 0.39, respectively. Meanwhile, NR, GS, and GOGAT together explained 88% and 89% of the variation in SP and TN, respectively. Among them, GOGAT had a greater effect on SP, with a standardized path coefficient of 0.81; GS had a greater effect on TN, with a standardized path coefficient of 0.72. Irrigation amount (W) and nitrogen application rate (N) explained 41%, 66%, and 61% of the variation in NR, GS, and GOGAT, respectively. Among them, W and N had greater effects on GS, with standardized path coefficients of 0.73 and 0.36, respectively. Furthermore, the total standardized effects of W, N, NR, GS, GOGAT, SP, TN, and yield on CP-a were 0.653, 0.333, −0.170, 0.609, 0.442, 0.457, 0.526, and −0.390, respectively (Figure 10b).

#### 2.4.2. Comprehensive Analysis

**Figure 11:**
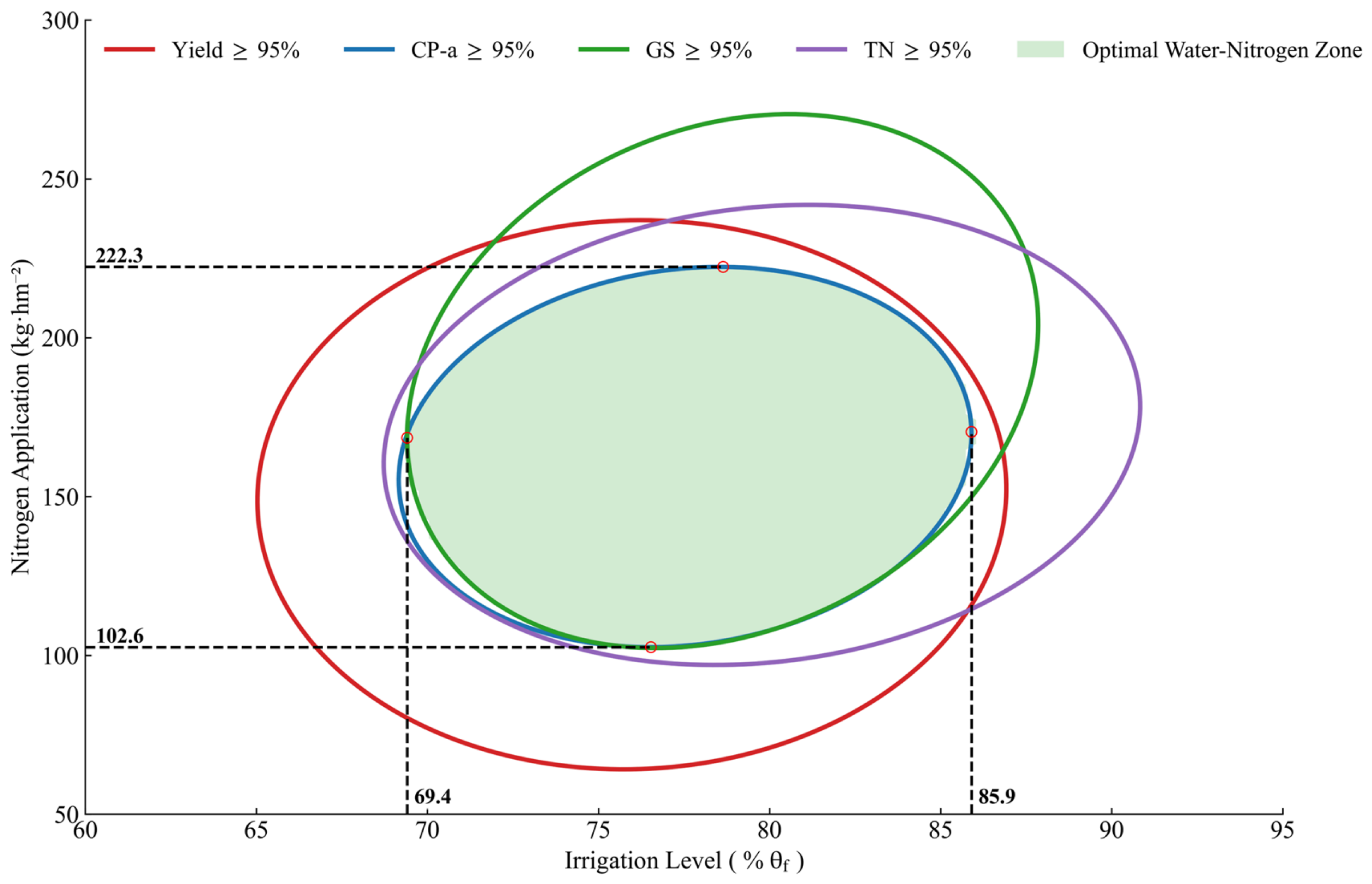
Regression model of GS-TN-Yield-CP-a in alfalfa under water–nitrogen regulation. Note: GS: glutamine synthetase; TN: total plant nitrogen content; Yield; CP-a: crude protein accumulation. The X-axis represents irrigation amount (%θ_f_), and the Y-axis represents nitrogen application rate (kg·hm^−2^). The red solid line indicates the contour boundary at which yield reaches 95% of its maximum value; the blue solid line indicates the contour boundary at which crude protein accumulation reaches 95% of its maximum value; the green solid line indicates the contour boundary at which glutamine synthetase reaches 95% of its maximum value; and the purple solid line indicates the contour boundary at which total plant nitrogen content reaches 95% of its maximum value. The light green shaded area (Optimal Water–Nitrogen Zone) represents the overlapping region where all four indicators simultaneously reach ≥95% of their maximum values.

The SEM results showed that irrigation amount (W) and nitrogen application rate (N) had significant positive regulatory effects on glutamine synthetase (GS) activity, with path coefficients of 0.73 and 0.36, respectively. Among the nitrogen metabolism-related product indicators, GS had a highly significant positive effect on TN, with a path coefficient of 0.72. Therefore, GS was selected as the key indicator of nitrogen metabolism-related enzymes, and TN was selected as the key indicator of nitrogen metabolism-related products. A regression model of GS-TN-Yield-CP-a was constructed by combining yield and crude protein accumulation (CP-a). Meanwhile, 95% of the maximum value of each of the four indicators was taken as the optimization condition, and they were superimposed to construct a multi-indicator overlapping contour plot (Figure 11). The regression model of GS-TN-Yield-CP-a showed that under water–nitrogen regulation, the optimal irrigation range for achieving high quality and high yield of alfalfa was 69.4–85.9%θ_f_, and the optimal nitrogen application range was 102.6–222.3 kg·hm^−2^.

## 3. Discussion

### 3.1. Effects of Water and Nitrogen Regulation on the Activity of Enzymes Involved in Nitrogen Metabolism

Water and nitrogen are key factors for plant growth, and nitrogen metabolism influences nitrogen uptake and utilization in alfalfa by regulating the activities of nitrate reductase (NR), glutamine synthetase (GS), and glutamate synthase (GOGAT). In this study, both irrigation amount and nitrogen application rate significantly affected the activities of NR, GS, and GOGAT in alfalfa leaves (*p* < 0.05), indicating that water and nitrogen conditions jointly regulate the nitrogen metabolism process in alfalfa. Meanwhile, under the same irrigation level, leaf NR activity first increased and then decreased with increasing nitrogen application rate, reaching its maximum at the N2 level, which was on average 26.85% higher than that at the N0 level. This finding is consistent with that of Ihsan et al. [19], and the possible reason is that NR, as the initial key enzyme in nitrogen assimilation, reduces NO_3_^−^ to NH_4_^+^ [20]. At the same time, moderate nitrogen application can increase the available nitrogen supply in the soil, enhance the leaf demand for nitrate–nitrogen utilization, and thus promote NR activity. When water is insufficient or under excessive nitrogen application, excessive nitrate–nitrogen accumulation in the plant leads to an imbalance between nitrogen uptake and utilization. Meanwhile, rapid plant growth under high nitrogen conditions may also accelerate leaf senescence, ultimately leading to a decline in NR activity [21]. Therefore, leaf NR activity in alfalfa exhibits a certain responsive range to nitrogen application levels, and appropriate nitrogen supply is more favorable for maintaining higher nitrate reduction capacity and normal plant growth [22]. Tang et al. [23] found in their study on the effects of water–nitrogen management on root morphology, nitrogen metabolism, and yield of rice that under the same nitrogen application rate, mild water deficit increased GS and GOGAT activities, while severe water deficit significantly reduced enzyme activities. This is consistent with the findings of the present study, indicating that mild water deficit can stimulate adaptive responses in plants, promote root nutrient uptake and internal substance transport, and thereby enhance related enzyme activities. However, as water deficit intensifies, leaf water loss and stomatal closure limit photosynthesis and energy supply, while cellular metabolism is inhibited, ultimately leading to a decline in GS and GOGAT activities [24]. Shi et al. [25] found in their study on the effects of different nitrogen treatments on the growth and nitrogen metabolism of ryegrass seedlings that under the same nitrogen application rate, GS activity exhibited a unimodal trend of first increasing and then decreasing, while GOGAT activity showed a bimodal trend of first increasing, then decreasing, then increasing again and decreasing again. This is not consistent with the findings of the present study. A possible reason is that alfalfa, as a leguminous nitrogen-fixing plant, can perform biological nitrogen fixation through root nodules, thereby making its nitrogen metabolism process more stable [26]. Furthermore, this study also found that NR, GS, and GOGAT activities all first increased and then decreased with increasing irrigation amount and nitrogen application rate, reaching their maxima under mild water deficit with a nitrogen application rate of 160 kg·hm^−2^. This is consistent with the findings of Luo et al. [27]. This may be because moderate water deficit promotes root uptake and transport of nitrogen, while medium nitrogen application can enhance the synthesis and assimilation of nitrogen metabolism enzymes, and both factors jointly promote the maintenance of nitrogen metabolism-related enzyme activities. However, excessive water or nitrogen supply may lead to nitrogen surplus, which inhibits the activity of nitrogen metabolism enzymes [28]. Therefore, appropriate water–nitrogen combinations can optimize nitrogen metabolism and nitrogen use efficiency in alfalfa, thereby enhancing enzyme activities.

### 3.2. Effects of Water and Nitrogen Regulation on Nitrogen Metabolism-Related Products

Soluble protein (SP) is one of the important osmotic regulatory substances in plants, and its content changes can directly reflect the nitrogen metabolism level and stress response capacity of plants [29]. In this study, leaf SP content of alfalfa first increased and then decreased with the progression of growth stages, reaching its peak at the budding stage, indicating that the plant material metabolism was most active and protein synthesis capacity was strongest at the budding stage. After entering the flowering stage, photosynthetic products and nitrogen gradually translocated to reproductive organs, leaf protein decomposition accelerated, and SP content subsequently decreased [30]. Appropriate water–nitrogen combinations had highly significant effects on SP content (*p* < 0.01). This study found that under the same nitrogen application rate, SP content increased with increasing irrigation amount, which is consistent with the findings of Chi et al. [31]. A possible reason is that adequate water conditions favor root uptake and transport of nitrogen, providing sufficient nitrogen sources for protein synthesis, thereby increasing SP content [32]. However, the promoting effect of water on SP accumulation is still influenced by nitrogen application level; only when nitrogen supply meets the demand of plants can improved water availability further promote its accumulation. Cai et al. [33] and Li et al. [34] found that under the same irrigation level, SP content first increased and then decreased with increasing nitrogen application rate, which is consistent with the findings of the present study, indicating that within a certain range, moderate nitrogen application can significantly increase SP content in alfalfa, while excessive nitrogen application may produce inhibitory effects [35].

Aboveground nitrogen content reflects the level of nitrogen uptake and accumulation in crops and serves as a key indicator linking exogenous nitrogen supply with internal nitrogen metabolism processes [36]. In this study, leaf nitrogen content was consistently higher than stem nitrogen content, which is consistent with the findings of Chen et al. [37]. A possible reason is that leaves are the primary sites of nitrogen assimilation, containing abundant functional proteins and requiring greater nitrogen supply, while stems mainly function in support and transport, with relatively higher structural carbohydrate content and lower nitrogen demand [38]. Zhang et al. [39] found in their study on water–nitrogen interaction on carbon and nitrogen accumulation in summer maize that under the same irrigation level, nitrogen content in various organs increased continuously with increasing nitrogen application rate, reaching its maximum at N300. This is not consistent with the findings of the present study. A possible reason is that under potted conditions, the restricted root-zone space may lead to excessive nutrient concentration or ionic imbalance at high nitrogen levels, thereby reducing nitrogen use efficiency, whereas this effect is weaker in field experiments or those with better root extension conditions [40]. Furthermore, Zhang et al. [41] found that nitrogen accumulation in tomato organs first increased and then decreased with increasing irrigation level, peaking at W2, which is consistent with the findings of the present study. This indicates that appropriate irrigation can promote nitrogen uptake, transport, and accumulation in plants, while excessively high or low water conditions may limit nitrogen use efficiency and thus hinder nitrogen accumulation [42].

### 3.3. The Effects of Water and Nitrogen Regulation on Production Performance

Appropriate irrigation and fertilization are important measures for achieving high crop yield. Studies have found that under the same irrigation level, dry matter yield of tomato at maturity first increased and then decreased with increasing nitrogen application rate [43]. Similarly, apple yield also first increased and then decreased with increasing irrigation amount and nitrogen application rate, with a clear threshold effect [44]. This study also found that under the same irrigation level, alfalfa yield first increased and then decreased with increasing nitrogen application rate, and the two-year average yield reached its maximum under the W2N2 treatment, which was 7.88% higher than that under W2N3. This indicates that when the nitrogen application rate is within the appropriate range, it can effectively promote leaf photosynthetic efficiency and dry matter accumulation. However, once this threshold is exceeded, the excess nitrogen cannot be converted into biomass; instead, it causes soil salt osmotic stress or intracellular ammonium toxicity, aggravating the metabolic burden of the plant, ultimately leading to yield reduction under high nitrogen treatments [45]. Furthermore, this study found that under the same nitrogen level, alfalfa yield first increased and then decreased with increasing irrigation amount, reaching its maximum at the W2 level, following the order W2 > W3 > W1 > W0. This is consistent with the findings of Liu et al. [46]. A possible reason is that the regulatory effect of water on alfalfa yield is influenced by nitrogen level. Appropriate water conditions can enhance nitrogen uptake and dry matter accumulation, while excessive irrigation can lead to soil water saturation and pore closure, causing severe hypoxia around the root system, thereby inhibiting root activity and ultimately limiting further accumulation of aboveground dry matter [47]. Li et al. [48] found that wheat yield increased with increasing irrigation and nitrogen application rates, which is inconsistent with the findings of the present study. This may be because wheat, as an annual grass crop, is highly responsive to external water and nitrogen inputs for yield formation, and increased water and nitrogen supply can continuously promote biomass and grain production. In contrast, alfalfa is a perennial leguminous forage that possesses symbiotic nitrogen fixation via root nodules, and excessive water and nitrogen inputs may impose negative feedback inhibition on key enzymes involved in nitrogen metabolism and on nitrogen fixation efficiency [49].

Nitrogen is an important constituent element of protein synthesis, and crude protein content in plants largely reflects the status of nitrogen uptake and nutrient accumulation [50]. Ma et al. [51] found that under the same irrigation level, crude protein content in alfalfa first increased and then decreased with increasing nitrogen application rate, which is consistent with the findings of the present study. This may be because moderate nitrogen application can provide sufficient nitrogen sources for protein synthesis, promote the accumulation of nitrogenous compounds in plants, and thereby increase crude protein content. However, excessive nitrogen application may disrupt the carbon–nitrogen balance in plants, making it difficult for a portion of the absorbed nitrogen to be effectively converted into protein, ultimately leading to a decline in crude protein content [52]. This study found that under the same nitrogen level, crude protein content of alfalfa first increased and then decreased with increasing irrigation amount, which is consistent with the findings of Jiu et al. [53]. This indicates that crude protein formation depends on the coordinated supply of water and nutrients, and appropriate soil moisture conditions can promote the accumulation of nitrogenous compounds and their conversion into protein in plants, thus favoring the improvement of crude protein content [54]. Furthermore, this study also found that crude protein accumulation was significantly influenced by irrigation amount and nitrogen application rate, reaching its maximum under the W2N2 treatment. This may be because appropriate water–nitrogen supply can simultaneously promote plant growth and protein accumulation, enabling synergistic improvements in yield and crude protein content, thereby increasing protein output per unit area. However, excessively high or low water–nitrogen levels may inhibit biomass formation or reduce protein accumulation capacity, ultimately leading to a decrease in crude protein accumulation [55].

This study was based on a two-year pot experiment and systematically analyzed the effects of water–nitrogen regulation on nitrogen metabolism and productive performance of alfalfa, and elucidated the interrelationships among various indicators using structural equation modeling. However, there are still certain differences between pot conditions and actual field production environments, and the experiment was conducted over only two years. Therefore, the long-term effects of water–nitrogen regulation and its applicability under different ecological conditions still require further verification. Future studies can combine long-term field experiments across multiple years and further consider the effects of different ecological environments, soil conditions, and climatic factors on the effectiveness of water–nitrogen regulation. Meanwhile, physiological, biochemical, and molecular indicators could be incorporated to further elucidate the underlying mechanisms by which water–nitrogen regulation affects nitrogen metabolism and productive performance in alfalfa, thereby providing a more reliable theoretical basis for regional efficient water and nitrogen management of alfalfa.

## 4. Materials and Methods

### 4.1. Overview of the Experimental Site

**Figure 12:**
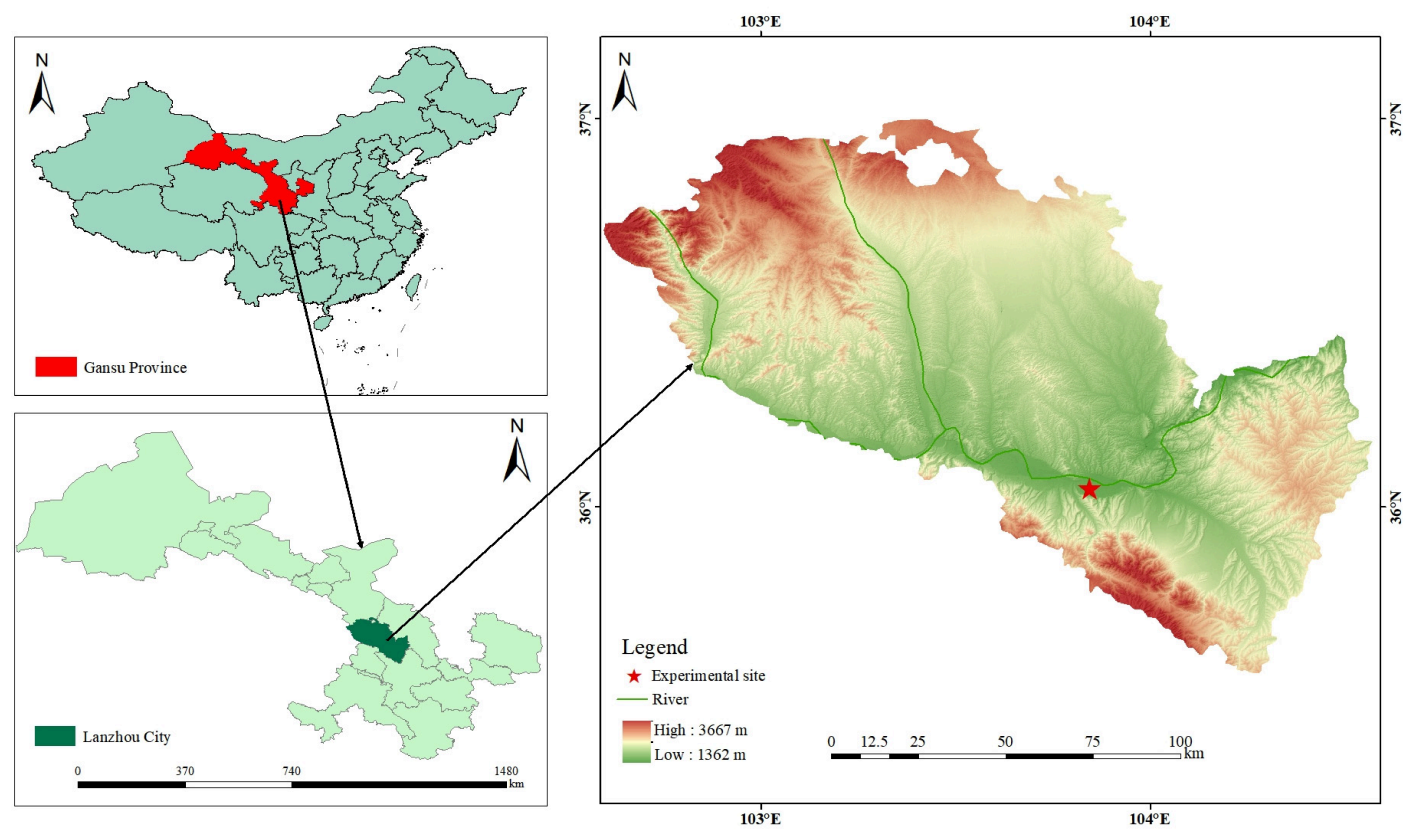
Overview map of the study area.

The experiment was conducted at the Teaching and Research Practice Base of the College of Grassland Science, Gansu Agricultural University (103°40′ E, 36°03′ N) in 2024 and 2025 (Figure 12). The experimental site is characterized by a temperate semi-arid continental climate, with a mean elevation of 1525 m, an annual mean temperature of 10.3 °C, a frost-free period of 108 days, an annual precipitation of 400–600 mm, an annual mean evaporation of 1410 mm, and an annual sunshine duration of 2100–2600 h. The soil type is loessial soil (Huangmian soil). The physicochemical properties of the 0–20 cm soil layer are detailed in Table 1.

### 4.2. Experimental Design

The experiment was conducted in a rain-sheltered greenhouse without temperature control, with both sides left open for ventilation. The experiment was conducted from April to September in both 2024 and 2025. The alfalfa variety used was “Gannong No.3” (*Medicago sativa* cv. Gannong No. 3, hereafter referred to as alfalfa), and the seeds were provided by the College of Grassland Science, Gansu Agricultural University. It possesses strong stress tolerance, high productive capacity, and stable forage quality, and is thus widely cultivated in the arid and semi-arid regions of northwestern China due to its excellent comprehensive performance [56]. The experiment had two factors: irrigation and nitrogen, each at four levels. Irrigation levels were based on soil water content relative to field capacity (θ_f_). Full experimental details are given in Table 2. Each irrigation treatment was defined by the controlled range of soil water content in the 0–20 cm layer; when the soil water content dropped to the lower limit of the corresponding range, irrigation was applied to bring it back to the upper limit to maintain different water gradients. The experiment adopted a randomized complete block design, comprising 16 treatments with three replications each, totaling 48 pots.

The pots were buried in the soil to approximately two-thirds of their height to simulate field conditions under different water–nitrogen combinations. PVC pipes (30 cm in diameter, 60 cm in height) were used as pots, each filled with 36 kg of a soil mixture prepared in a 5:1 ratio of native soil from the experimental site (yellow loam) to commercial seedling growing medium (Xinxiang Luyuan Seedling Growing Medium). The seedling medium used consisted primarily of peat, vermiculite, and perlite. Seeding was conducted on April 8, 2024; prior to seeding, the experimental site was tilled, leveled, and cleared of weeds. Forty seeds were broadcast evenly into each pot. When the alfalfa seedlings reached the three-leaf stage, manual thinning was performed, retaining 30 uniformly growing, healthy seedlings per pot. Before sowing, 40% of the total nitrogen fertilizer (urea, N ≥ 46.6%), together with calcium superphosphate at 3.09 g·pot^−1^ (16% P_2_O_5_) and potassium sulfate at 0.47 g·pot^−1^ (50% K_2_O), was applied as basal fertilizer in a single application [57]. The remaining 60% of the nitrogen fertilizer was topdressed in equal amounts at the regreening stage of each alfalfa cut. Details of the irrigation and nitrogen fertilization regimes are presented in Table 3.

The volumetric water content (VWC) of the 0–20 cm soil layer was measured every three days using a portable time-domain reflectometer (TRIME-PICO-IPH, IMKO, Ettlingen, Germany), with monitoring frequency increased during hot periods. When the monitored VWC of the pots dropped below the lower limit of the irrigation level of the respective treatment, manual irrigation was immediately performed. The required irrigation volume for each pot was calculated as I = (θ_u_ − θ_c_) × V_s_, where θ_u_ is the upper VWC target (%), θ_c_ is the current monitored VWC (%), and V_s_ is the effective soil volume (cm^3^). To accurately monitor and control the water applied, the calculated amount of water was precisely measured using a graduated cylinder and manually applied evenly to each pot. The exact volume of water applied for each irrigation event was logged. To ensure measurement accuracy and consistency in water control, the oven-drying method was adopted every seven days for supplementary verification of the soil moisture status. Alfalfa was harvested at the flowering stage of each cut. The first cut was conducted on 28 June 2024 and 3 July 2025; the second cut was conducted on 24 August 2024 and 8 September 2025.

### 4.3. Test Items and Methods

#### 4.3.1. Activity of Enzymes Involved in Nitrogen Metabolism

Sampling was conducted at the branching, budding, and flowering stages of each alfalfa cut. Three pots with healthy and uniformly growing alfalfa plants were selected from each treatment, and fully expanded and functional leaves from the upper and middle parts of the plants, free from pests and diseases, were randomly collected from each pot. After sampling, the leaves were immediately placed in liquid nitrogen and subsequently stored in an ultra-low temperature freezer at −80 °C for determination of nitrogen metabolism-related enzyme activities. The activities of nitrate reductase (NR), glutamine synthetase (GS), and glutamate synthase (GOGAT) were determined using assay kits (provided by Suzhou Grace Biotechnology Co., Ltd., Suzhou, China), and the definitions of enzyme activity units followed those specified in the kit instructions.

#### 4.3.2. Soluble Protein Content

Using the same leaf samples as those used for the determination of nitrogen metabolism-related enzyme activities, leaf soluble protein (SP) content was determined by the Coomassie Brilliant Blue method [58].

#### 4.3.3. Nitrogen Content and Accumulation in the Aboveground Parts

At the flowering stage of each alfalfa cut, ten uniformly growing alfalfa plants were randomly selected from each treatment and harvested. The stems and leaves were separated, placed in an oven at 105 °C for 30 min to deactivate enzymes, and then dried at 75 °C to constant weight. After cooling, the dry weight was recorded. The dried stems and leaves were ground using a small grinder and passed through a 1 mm sieve. Following digestion with H_2_SO_4_-H_2_O_2_, the nitrogen content (%) was determined using a FOSS 2300 automatic Kjeldahl apparatus [59].

Alfalfa leaf (stem) nitrogen accumulation (kg·ha^−1^) = leaf (stem) nitrogen content (%) × Dry weight of leaves (stems) (t·ha^−1^). The sum of the total nitrogen accumulated in the leaves and stems equals the total nitrogen accumulated by the alfalfa plant.

#### 4.3.4. Yield

At the flowering stage of each alfalfa cut, the plants were cut at 5 cm above the soil surface in the pot and weighed to obtain fresh weight. The fresh samples were then placed in an oven at 105 °C for 30 min for enzyme inactivation, and then dried at 75 °C for 48 h to constant weight. After cooling to room temperature, the dry weight (g·pot^−1^) was recorded and calculated as yield per hectare (Yield, t·ha^−1^).

#### 4.3.5. Crude Protein Content and Accumulated Amount

(1) Crude protein content

The calculation formula is as follows:

(1) CP (%) = TN (%) × 6.25

where CP is the crude protein content, TN is the total nitrogen content of alfalfa plants, and 6.25 is the protein conversion factor.

(2) Crude protein accumulation

The crude protein accumulation of alfalfa plants was calculated by multiplying yield by crude protein content. The calculation formula is as follows:

CP-a = Yield × CP/100(2)

where CP-a is the crude protein accumulation (kg·ha^−1^).

### 4.4. Data Analysis

Data were analyzed using Excel 2019 (Microsoft Corporation, Redmond, WA, USA). Analysis of variance (ANOVA) was performed using SPSS 25.0 (IBM Corporation, Chicago, IL, USA). Prior to analysis of variance (ANOVA), data normality was verified using the Shapiro–Wilk test, and homogeneity of variance was tested using Levene’s test. One-way ANOVA was then conducted, and multiple comparisons among treatment means were performed using the least significant difference (LSD) test at a significance level of *p* < 0.05. Graphs were plotted using Origin 2024b (OriginLab Corporation, Northampton, MA, USA). Structural equation modeling (SEM) was constructed in Amos 28 (IBM Corporation, Armonk, NY, USA) based on the covariance matrix among variables, and the path coefficients of the structural equation model were estimated using the maximum likelihood estimation method. The goodness of fit of the SEM was evaluated using the relative chi-square value (χ^2^/df), goodness-of-fit index (GFI), comparative fit index (CFI), and root mean square error of approximation (RMSEA). To minimize the effects of inter-annual and inter-cut variations, the activities of nitrogen metabolism-related enzymes and soluble protein (SP) content used in statistical analysis were the mean values measured at the corresponding growth stages across the two years and all cuts, while the other indicators were the mean values of measurements taken across the two years and all cuts.

## 5. Conclusions

(1) Mild water deficit irrigation combined with nitrogen application at 160 kg·hm^−2^ (W2N2) significantly promoted the activities of nitrogen metabolism-related enzymes in alfalfa, enhanced nitrogen accumulation, and subsequently promoted the formation of yield and crude protein accumulation.

(2) Both irrigation amount and nitrogen application rate significantly affected SP content, aboveground nitrogen content, and nitrogen accumulation in alfalfa (*p* < 0.05). Appropriate water–nitrogen supply promoted the accumulation of nitrogen metabolites and improved plant nitrogen nutrition, with leaves being the primary site of aboveground nitrogen accumulation.

(3) Appropriate water–nitrogen treatment combinations synergistically improved alfalfa yield and quality. Under W2N2, alfalfa yield, crude protein content (CP), and crude protein accumulation (CP-a) all reached their maximum values, averaging 10.22 t·ha^−1^, 23.13%, and 1187.10 kg·ha^−1^, respectively.

Through screening key indicators by SEM and constructing a comprehensive evaluation model, it was found that under water–nitrogen regulation, the optimal irrigation range for achieving high quality and high yield of alfalfa is 69.4–85.9% θ_f_, and the optimal nitrogen application range is 102.6–222.3 kg·hm^−2^. These results are in good agreement with the superior productive performance observed under the W2N2 treatment in the experiment, and can preliminarily provide a theoretical basis and technical reference for high-yield and high-quality alfalfa cultivation and water–nitrogen management in arid and semi-arid regions. Future studies should combine field experiments in different ecological regions to further verify and optimize the proposed suitable water–nitrogen regulation ranges and the comprehensive evaluation model.

## Figures and Tables

**Table 1:** Experimental soil physicochemical properties.

Property	Field Capacity (cm^3^·cm^−3^)	Bulk Density (g·cm^−3^)	pH	Organic Matter (g·kg^−1^)	Total N (g·kg^−1^)	Available N (mg·kg^−1^)	Available P (mg·kg^−1^)	Available K (g·kg^−1^)
Value	28.2	0.97	7.7	16.58	0.28	68.83	3.68	0.22

**Table 2:** Experimental design scheme.

Treatment	Irrigation Level (θ_f_)		Nitrogen Application (kg·hm^−2^)
W0N0	Severe water deficit	45–60%	0
W0N1			80
W0N2			160
W0N3			240
W1N0	Moderate water deficit	55–70%	0
W1N1			80
W1N2			160
W1N3			240
W2N0	Mild water deficit	65–80%	0
W2N1			80
W2N2			160
W2N3			240
W3N0	Full irrigation	75–90%	0
W3N1			80
W3N2			160
W3N3			240

**Table 3:** Irrigation and nitrogen fertilization regimes in the experiment.

Treatment	Total Irrigation Quota (mm)	Base Fertilizer (kg·hm^−2^)	Total Topdressing Fertilizer (kg·hm^−2^)
W0N0	239	0	0
W0N1	239	32	48
W0N2	239	64	96
W0N3	239	96	144
W1N0	282	0	0
W1N1	282	32	48
W1N2	282	64	96
W1N3	282	96	144
W2N0	326	0	0
W2N1	326	32	48
W2N2	326	64	96
W2N3	326	96	144
W3N0	427	0	0
W3N1	427	32	48
W3N2	427	64	96
W3N3	427	96	144

## Data Availability

All data are incorporated into the article.
